# Health literacy and falls among community-dwelling older people in China: is there a sex difference?

**DOI:** 10.1007/s40520-024-02788-6

**Published:** 2024-07-18

**Authors:** Shaojie Li, Jingjing Wang, Longbing Ren, Pengpeng Ye, Wenyi Niu, Mingzhi Yu, Yang Hu, Yuling Jiang, Yifei Wu, Maoyi Tian, Yali Zhao, Yao Yao

**Affiliations:** 1 School of Public Health, Peking University, Beijing, China; 2 China Center for Health Development Studies, Peking University, Beijing, China; 3 National Centre for Non-Communicable Disease Control and Prevention, Chinese Centre for Disease Control and Prevention, Beijing, China; 4 School of Public Health, Harbin Medical University, Harbin, 150081 China; 5 Department of General Practice, The Second Affiliated Hospital of Harbin Medical University, Harbin, China; 6 Central Laboratory, Hainan Hospital of Chinese People’s Liberation Army General Hospital, Sanya, China; 7 Key Laboratory of Epidemiology of Major Diseases (Peking University), Ministry of Education, Beijing, China

**Keywords:** Health literacy, Falls, Sex difference, Cross-sectional study

## Abstract

**Background:**

Health literacy is one of the important determinants of healthy aging, yet few studies have focused on the association between health literacy and falls.

**Aims:**

This study aims to explore the relationship between health literacy and falls, with a focus on sex differences among older people in China.

**Methods:**

This cross-sectional study enrolled 2,144 older people aged ≥ 60 years from Shandong Province, China in 2021. We used general health literacy screening scale to assess health literacy, and collected the incidence of falls in the past year. Logistic regression models were employed to analyze the relationship between health literacy and falls. We investigated the sex differences by subgroup analyses.

**Results:**

The prevalence of adequate health literacy and falls was 21.7% (95% CI: 20.0–23.5%) and 25.4% (95% CI: 23.6–27.3%), respectively. In a fully-adjusted model, adequate health literacy was associated with a lower prevalence of falls in older adults (OR = 0.71, 95%CI: 0.52–0.96). Subgroup analysis revealed sex differences in this relationship (*P*_for interaction_ <0.05). Specifically, the female group showed no significant relationship between health literacy and falls (OR = 0.92, 95% CI: 0.59–1.44); however, the male group demonstrated a robust and significant relationship (OR = 0.58, 95% CI: 0.37–0.90).

**Conclusions:**

Older people with adequate health literacy have lower prevalence of falls, which appears to differ by sex. This relationship was significant among men but not among women. These findings emphasize the need for policymakers and healthcare providers to consider sex differences when designing and implementing programs aimed at improving health literacy and preventing falls in the older population. Improving health literacy among older women could be a strategic component in bridging sex inequality in falls.

**Supplementary Information:**

The online version contains supplementary material available at 10.1007/s40520-024-02788-6.

## Introduction

Falls refer to an unexpected event in which the individual comes to rest on the ground, floor or a lower level without a known loss of consciousness [1]. More than a quarter of older people worldwide experienced a fall per year [2] and falls were an important cause of injury-related deaths in older people [3]. Notably, World Health Organization (WHO) estimates showed that more than 80% of fall-related deaths globally occur in low- and middle-income countries (LMICs), indicating an urgent need for prevention in LMICs [4]. Meanwhile, the high prevalence of falls has resulted in several detrimental conditions for older people in their later life, such as fractures [5], disabilities [6], and higher costs and demands for care.

Identifying modifiable risk factors for falls is an important basis for falls prevention. Previous meta-analyses have explored numerous risk factors associated with falls, such as balance disorder, substance use, depressive symptoms, and visual impairment [7]. Based on these findings, many scholars have conducted multidisciplinary and multifactorial fall intervention studies, such as strength and balance training and environmental modifications [8, 9]. Although these interventions have been shown to be effective [8, 9], it is worth noting that strength and balance training mostly requires professional guidance, and home environment modifications are often financially costly and may have limitations when it comes to widespread replication. Therefore, there remains a need to explore the factors associated with falls in older adults to inform the development of convenient and cost-effective intervention strategies. Improving the health literacy of the global population is cited by WHO as one of the key tasks of health promotion [10].

Health literacy refers to the ability of individuals to access, process and understand health information and services, and to make health decisions [11]. Previous studies have demonstrated that low health literacy is associated with the occurrence of multiple adverse health events in older adults, such as frailty [12] and dysphagia [13]. In addition, a previous intervention study found that a health literacy intervention significantly improved glycemic control outcomes and renal function in older adults with type 2 diabetes [14]. These studies suggest that high health literacy may have many health benefits for older adults. However, few studies have focused on the association between health literacy and falls in older adults. Based on our literature search, there are only two empirical studies that have explored the association between health literacy and falls [15, 16]. The two studies, which focused on hospitalized older people [15] and community-dwelling older people [16], respectively, yielded opposite results. Specifically, the study of hospitalized older adults did not find health literacy to be associated with falls [15], whereas the study of community-dwelling older adults found the number of falls decreased with higher health literacy, albeit it was more pronounced among men [16]. It is worth noting that this study among community-dwelling older adults only compared and trend-analyzed the mean number of falls across health literacy subgroups, without controlling for potential confounders [16]. During our investigation into gray literature, we found two Chinese theses involving health literacy and falls among community-dwelling older individuals [17, 18]. However, these studies did not find a significant correlation between health literacy and the incidence of falls. Therefore, it remains unclear whether health literacy is associated with falls among community-dwelling older adults. A recent study proposed a multicomponent theoretical model on health literacy and concerns about falling, which suggests that health literacy may be closely associated with determinants relating to concerns about falling [19]. However, there is currently a lack of observational research evidence to support this. In addition, previous studies have found significant sex differences in health literacy levels [20] and fall incidence [21]. Compared to older men, older women often have lower health literacy and a higher incidence of falls [20, 21]. It may be inferred that there are also sex differences in the association between health literacy and falls, but this has not yet been explored. Clarifying these sex differences can aid in developing more targeted prevention and intervention strategies for falls, thereby reducing sex inequalities in fall incidents.

Currently, 97% of older individuals in China reside in the community [22]. According to data from a nationally representative survey, the incidence of falls among community-dwelling older adults was 22.2% in China [23]. Given the substantial size of this demographic, examining the relationship between health literacy and falls could offer valuable scientific insights to mitigate the disease burden associated with falls in community-dwelling older population. Therefore, to fill above literature gap, this study aims to explore the relationship between health literacy and falls, and its sex difference among community-dwelling older people in China, which may help provide scientific evidence for preventing falls in older people from a health literacy perspective.

## Methods

### Study design and participants

The data for this study was obtained from a cross-sectional survey conducted in Jinan City, Shandong Province from March to May 2021, and a total of 2,144 community-dwelling older people aged ≥ 60 years were included with a stratified cluster random sampling method. The sampling and recruitment process of the sample has been reported in detail in previous studies [24, 25]. The inclusion criteria for participants were as follows: individuals aged 60 and above, residing in Jinan for over 6 months, providing informed consent, and willing to participate this study. The exclusion criteria were: presence of hearing or language disorders; severe diseases such as malignant tumors, dementia, or schizophrenia; and disability. In the initial phase of this study, 2,557 participants who met the inclusion criteria were selected, of which 356 were excluded based on the exclusion criteria. During the survey, 57 participants withdrew. Consequently, 2,144 participants were included in the final analysis, with all individuals having complete data and no missing information. All surveys were conducted face-to-face using paper questionnaires by interviewers who had undergone standardized training. More information regarding sample recruitment and survey administration is presented in the supplementary materials.

We used the sample size formula for estimating overall rates for sample estimation [26]: $\text{n}=\frac{{{\text{Z}}_{{\alpha }/2}}^{2}\text{P}(1-\text{P})}{{{\delta }}^{2}}$. A meta-analysis including 54 studies showed that the prevalence of falls among Chinese older people was 19.3% [27]. In this study, we set α to 0.05, *P* to 0.193, Z_α/2_ to 1.96 and δ to 0.02, and resulted in a minimum sample size of 1496. The sample size of this study meets the minimum sample size requirement. The Medical Ethics Committee of the Xiangya School of Public Health, Central South University approved this survey (No: XYGW-2020-101) and all the participants provided written informed consent.

### Measures

#### Health literacy

We used 10-items of health literacy screening scale to assess self-perception health literacy (the scale is displayed in the supplementary material). This scale was developed by Chi et al. [28], in Taiwan, China, and modified and validated by Li et al., in mainland China [29]. Each item is scored using the Likert 5-point scoring method, with scores ranging from 1 to 5 for “strongly disagree” to “strongly agree”. The sum of ten items is the total score, with a score range of 10–50 points. The higher the score, the better the health literacy. In this study, according to the health literacy classification criteria provided by the previous Chinese citizen health literacy monitoring [30] (≥ 80% score), we use 40 points as the threshold for dividing adequate and inadequate health literacy.

#### Falls

In alignment with previous studies [31], we asked older people about the incidence of falls in the past year by face to face household survey. The older people who answered “Yes” were considered to have fallen.

#### Covariates

In this study, we set up three sets of covariates: sociodemographic characteristics, lifestyles, and health-related status. Sociodemographic characteristics included age (60–79, 80+), sex (male, female), residence (urban, rural), marital status (unmarried, married), educational level (primary school and below, junior high school, high school and above), and average monthly personal income (< 2000, 2000–3000, > 3000, Chinese yuan [CNY]). Lifestyles included smoking status (no, yes), drinking status (no, yes), vegetable intake (hardly, regularly), fruit intake (hardly, regularly), and physical exercise (hardly, regularly). Health-related status included chronic disease (no, yes), self-rated health (poor, moderate, good), functional limitation (no, yes), and body mass index (BMI). Among them, we inquired whether participants had been diagnosed by a doctor with any chronic diseases, including hypertension, hyperlipidemia, hypercholesterolemia, diabetes, coronary heart disease, arthritis, cervical spondylosis, and chronic obstructive pulmonary disease. Having any one of these conditions was considered as the presence of a chronic disease. In addition, we used 6 activities of daily living (walking, eating, dressing, washing, bathing, and using the toilet) and 8 instrumental activities of daily living (taking a bus, cooking, doing household chores, taking medication, doing laundry, shopping, making telephone calls, and managing money) [32] to evaluate functional limitation. The presence of one or more above activities that cannot be completed independently is judged as having functional limitation. BMI is calculated based on self-reported height (m) and weight (kg), and is divided into four groups according to Chinese standards [33]: thin (< 18.5 kg/m^2^), normal (18.5–23.9 kg/m^2^), overweight (24.0–27.9 kg/m^2^), obesity (≥ 28.0 kg/m^2^).

### Statistical analyses

The description of health literacy, falls, and covariates were presented as frequencies (n) with percentages (%). We used the chi-squared test to assess the differences in covariates of the participants among health literacy and falls. We conducted logistic regression model to analyze the relationship between health literacy and falls. We established four logistic regression models based on the different covariates adjusted. Model 1 was the unadjusted model. Model 2 adjusted for sociodemographic characteristics, and model 3 adjusted for lifestyles based on model 2. Model 4 further adjusted for health-related status based on model 3. In addition, we performed a subgroup analysis to examine the disparity in the relationship between health literacy and falls according to sex. Meanwhile, to test the robustness of the above relationship, we conducted a sensitivity analysis by further adding depressive symptom, mild cognitive impairment, and self-rated balance impairment as covariates. The measurement of three covariates can be found in the supplementary materials. We used the odds ratio (OR) and 95% confidence interval (CI) to evaluate the strength and significance of above relationship. All analyses were used by STATA 17.0 (Stata Corp, College Station, TX, USA), and *P* < 0.05 indicated statistical significance.

## Results

### Descriptive statistics

Among the 2,144 participants (mean age: 72.0 ± 6.96 years; 1069 females [49.9%]), the rates of adequate health literacy and fall among older people were 21.7% (95%CI: 20.0–23.5%) and 25.4% (95%CI: 23.6–27.3%). Participant characteristics and their difference between groups according to health literacy and fall are shown in Table 1. More information on health literacy scores and subitem scoring by sex were presented in the supplementary materials.

**Table 1:** Characteristics of participants according to falls

Variables	Total	Health literacy		*P* value	Fall		
		Inadequate	Adequate		No	Yes	*P* value
Total sample	2144 (100.0)	1678 (78.3)	466 (21.7)		1599 (74.6)	545 (25.4)	
Age, years				0.013			< 0.001
60–79	1822 (85.0)	1409 (84.0)	413 (88.6)		1391 (87.0)	431 (79.1)	
≥ 80	322 (15.0)	269 (16.0)	53 (11.4)		208 (13.0)	114 (20.9)	
Sex				< 0.001			0.009
Male	1075 (50.1)	784 (46.7)	291 (62.4)		828 (51.8)	247 (45.3)	
Female	1069 (49.9)	894 (53.3)	175 (37.6)		771 (48.2)	298 (54.7)	
Residence				< 0.001			< 0.001
Urban residents	829 (38.7)	562 (33.5)	267 (57.3)		655 (41.0)	174 (31.9)	
Rural residents	1315 (61.3)	1116 (66.5)	199 (42.7)		944 (59.0)	371 (68.1)	
Marital status				< 0.001			< 0.001
Unmarried	498 (23.2)	431 (25.7)	67 (14.4)		333 (20.8)	165 (30.3)	
Married	1646 (76.8)	1247 (74.3)	399 (85.6)		1266 (79.2)	380 (69.7)	
Educational level				< 0.001			0.003
Primary school and below	509 (23.7)	471 (28.1)	38 (8.2)		356 (22.3)	153 (28.1)	
Junior high school	820 (38.2)	686 (40.9)	134 (28.8)		605 (37.8)	215 (39.4)	
High school and above	815 (38.0)	521 (31.0)	294 (63.1)		638 (39.9)	177 (32.5)	
Income (CNY)				< 0.001			< 0.001
< 2000	1053 (49.1)	930 (55.4)	123 (26.4)		719 (45.0)	334 (61.3)	
2000–3000	720 (33.6)	547 (32.6)	173 (37.1)		580 (36.3)	140 (25.7)	
> 3000	371 (17.3)	201 (12.0)	170 (36.5)		300 (18.8)	71 (13.0)	
Smoking status				0.290			< 0.001
No	1508 (70.3)	1171 (69.8)	337 (72.3)		1166 (72.9)	342 (62.8)	
Yes	636 (29.7)	507 (30.2)	129 (27.7)		433 (27.1)	203 (37.2)	
Drinking status				0.370			0.463
No	1788 (83.4)	1393 (83.0)	395 (84.8)		1339 (83.7)	449 (82.4)	
Yes	356 (16.6)	285 (17.0)	71 (15.2)		260 (16.3)	96 (17.6)	
Fruit intake				< 0.001			0.337
Hardly	977 (45.6)	837 (49.9)	140 (30.0)		719 (45.0)	258 (47.3)	
Regularly	1167 (54.4)	841 (50.1)	326 (70.0)		880 (55.0)	287 (52.7)	
Vegetable intake				< 0.001			0.200
Hardly	232 (10.8)	203 (12.1)	29 (6.2)		165 (10.3)	67 (12.3)	
Regularly	1912 (89.2)	1475 (87.9)	437 (93.8)		1434 (89.7)	478 (87.7)	
Physical exercise				< 0.001			< 0.001
Hardly	545 (25.4)	505 (30.1)	40 (8.6)		364 (22.8)	181 (33.2)	
Regularly	1599 (74.6)	1173 (69.9)	426 (91.4)		1235 (77.2)	364 (66.8)	
Chronic disease				< 0.001			< 0.001
No	635 (29.6)	458 (27.3)	177 (38.0)		533 (33.3)	102 (18.7)	
Yes	1509 (70.4)	1220 (72.7)	289 (62.0)		1066 (66.7)	443 (81.3)	
Self-rated health				< 0.001			< 0.001
Poor	451 (21.0)	410 (24.4)	41 (8.8)		244 (15.3)	207 (38.0)	
Moderate	702 (32.7)	592 (35.3)	110 (23.6)		512 (32.0)	190 (34.9)	
Good	991 (46.2)	676 (40.3)	315 (67.6)		843 (52.7)	148 (27.2)	
Functional limitation				< 0.001			< 0.001
No	1193 (55.6)	824 (49.1)	369 (79.2)		1003 (62.7)	190 (34.9)	
Yes	951 (44.4)	854 (50.9)	97 (20.8)		596 (37.3)	355 (65.1)	
Body mass index (kg/m^2^)				0.049			0.073
Thin (< 18.5)	179 (8.3)	152 (9.1)	27 (5.8)		119 (7.4)	60 (11.0)	
Normal (18.5–23.9)	1123 (52.4)	883 (52.6)	240 (51.5)		850 (53.2)	273 (50.1)	
Overweight (24.0–27.9)	666 (31.1)	503 (30.0)	163 (35.0)		499 (31.2)	167 (30.6)	
Obesity (≥ 28.0)	176 (8.2)	140 (8.3)	36 (7.7)		131 (8.2)	45 (8.3)	

Note: CNY: Chinese Yuan

### Association between health literacy and falls

The results of logistic regression model (Table 2) suggest that, in both the unadjusted and adjusted models, health literacy was all associated with falls in older people. Specifically, in model 1, older people with adequate health literacy were 57% less likely to have had a fall in the past year compared to those with inadequate health literacy. After adjusting for sociodemographic characteristics and lifestyles, respectively, the relationship between health literacy and falls was reduced but still statistically significant. In fully-adjusted model, we also found adequate health literacy is associated with a lower prevalence of fall in older people (OR = 0.71, 95%CI: 0.52–0.96).

**Table 2:** Relationship between adequate health literacy and falls

Model	OR	SE	95%CI of OR	*P*-value
Model 1	0.43	0.06	0.33–0.57	< 0.001
Model 2	0.53	0.08	0.39–0.71	< 0.001
Model 3	0.55	0.08	0.41–0.74	< 0.001
Model 4	0.71	0.11	0.52–0.96	0.028

Note: Model 1 was unadjusted model; Model 2 adjusted for sociodemographic characteristics; Model 3 adjusted for lifestyles based on model 2; Model 4 further adjusted for health status based on model 3

### Sex-stratified analysis

**Fig. 1:**
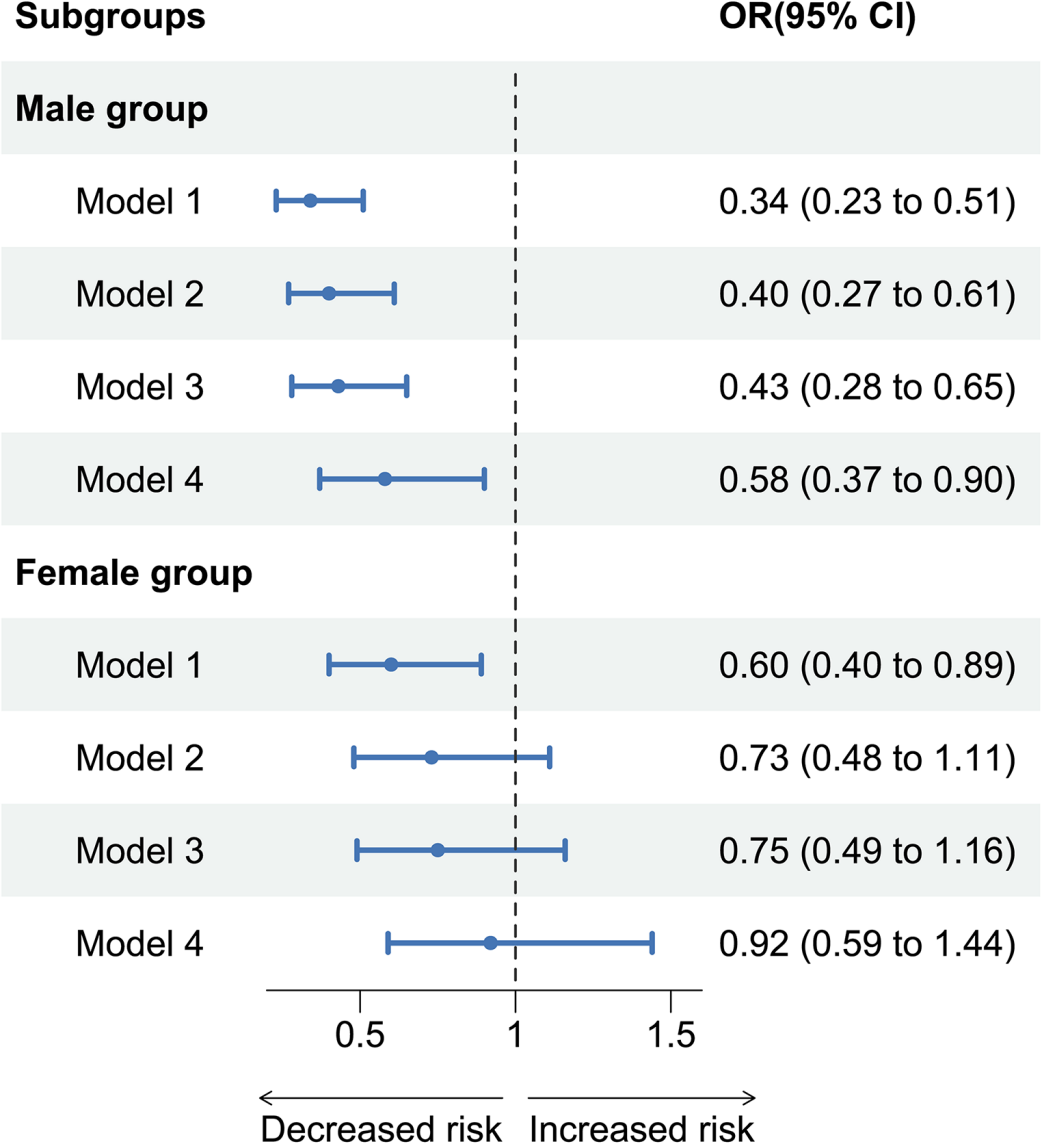
The sex difference in the relationship between adequate health literacy and falls. Note: Model 1 was unadjusted model; Model 2 adjusted for sociodemographic characteristics; Model 3 adjusted for lifestyles based on model 2; Model 4 further adjusted for health status based on model 3

Unadjusted and adjusted relationship between health literacy and falls in sex-stratified participants are shown in Fig. 1. In the unadjusted model, we found that health literacy was all associated with falls both in male and female groups. After adjusting covariates, there are sex differences in the above relationships (*P*_for interaction_ <0.05). Specifically, in fully-adjusted model, the female group showed no relationship between health literacy and falls (OR = 0.92, 95% CI: 0.59–1.44); however, the male group showed higher relationship (OR = 0.58, 95% CI: 0.37–0.90).

### Sensitivity analysis

The results of sensitivity analysis showed that after adjusting depressive symptom, mild cognitive impairment, and self-rated balance impairment, the association between health literacy and falls among older adults was still significant (OR = 0.72, 95%CI = 0.53–0.99). In addition, there are still sex differences in the above associations, which are more pronounced in males (OR = 0.52, 95%CI = 0.34–0.81) and not significant in females (OR = 0.90, 95%CI = 0.58–1.41).

## Discussion

To our knowledge, this study is the first to investigate the relationship between health literacy and falls among community-dwelling older people. In the present study, adequate health literacy was associated with a lower prevalence of falls after adjusting for potential confounders. In addition, this study found sex differences in above relationship, which were stronger in the male sample.

While a previous study has shown that health literacy is not significantly associated with falls among hospitalized older people [15], our study based on community-dwelling older people found the opposite to be true. In our study, older adults with adequate health literacy had a 29% lower prevalence risk of falls compared with those with inadequate health literacy. This study further adds to the risk factors associated with falls among older people and provides a new direction for conducting fall prevention and interventions. The mechanism of relationship between health literacy and falls may involve the following areas. First, health literacy has been recognized as an important determinant of health behaviors among older adults. The Behavioral Risk Factor Surveillance Study in the United States found that inadequate health literacy was associated with a wide range of poor health behaviors such as physical inactivity, sleep deprivation, and irregular health examination [34]. A systematic review and meta-analysis also found that low health literacy was significantly associated with insufficient physical activity among older people [35]. These unhealthy behaviors and lifestyles have been shown to be important risk factors for falls in previous studies [36, 37]. Second, lower health literacy is associated with decreased physical functioning [38], which may further increase the likelihood of falls occurring. A community-based study of older adults found a significant positive relationship between health literacy and gait speed [39], while low gait speed is associated with an increased risk of falls [40]. In addition, inadequate health literacy also increased the risk of adverse health events, such as frailty [41], psychological distress (depression, anxiety, and stress) [42], and obesity [43]. These adverse health events have been proven to increase the risk of falls in meta-analyses [44, 45]. Based on the above discussion, health literacy may be associated with falls in older people through pathways such as health behaviors, physical functioning, and adverse health events, which requires further exploration of the mechanisms in future studies.

Another important finding of this study is the sex difference in the relationship between health literacy and falls among community-dwelling older people. We found the above relationship to be significant only in males, but not in females. Research on sex differences in the distribution of health literacy and falls has been reported in several studies. In our study, the total score (33.90 ± 7.78) and subitem scores of male health literacy were significantly higher than those of females (30.74 ± 8.50), and the rate of adequate health literacy was also higher in males (27.1%) than in females (16.4%). A previous study of more than 7,000 community-dwelling older people in Taiwan, China, found that men’s health literacy levels were significantly higher than women’s [46]. The results of the Health Literacy Monitoring Survey of Chinese Residents show the same results [47]. These findings are all consistent with our results. Sex differences in health literacy may be related to educational attainment. Education is known to be a determinant of literacy and health literacy [48], and there are large sex inequalities in educational attainment among Chinese older people, with men having much higher educational attainment than women [49]. In addition, sex differences in falls in older people are also widely discussed [21]. Higher fall rates in women than in men may be related to differences in physical fitness and physical activity between men and women [50]. There may be several possible explanations for the sex differences in the relationship between health literacy and falls. First, men’s health literacy is significantly higher than women’s, which may lead men to be more concerned about their health status and engage in healthy lifestyles, such as avoiding smoking [51] and increasing physical activity [52], which may contribute to lowering the risk of falls. Secondly, there is a clear “male-female health-survival paradox” between men and women, whereby women usually live longer than men, but their health status is significantly lower than men’s, especially in later life [53]. The mechanisms behind this phenomenon are complex, with genetic, immune, metabolic, behavioral and socio-economic status [54]. In this scenario, despite the benefits of health literacy in promoting health status, its benefits on falls may not be significant in the context of socioeconomic characteristics, lifestyle and health status, resulting in a statistically non-significant relationship between health literacy and falls among older women. This is confirmed by our results that adequate health literacy is associated with low prevalence of falls among older women in the unadjusted model. However, when adjusted for sociodemographic characteristics, lifestyle, and health status, there was no statistical significance. This sex difference has also been confirmed in a previous study, that is, oral health literacy was significantly associated with oral health-related quality of life were in older men, not in older women [55].

Our study has important public health implications for promoting fall prevention among community-dwelling older people. Our study suggests that improving the health literacy of community-dwelling older people may help to reduce their prevalence of falls, which provides new strategies and directions for fall prevention. In addition, the observed sex differences in health literacy and fall among older adults underscore a significant public health concern. This disparity suggests that women may be at a higher fall risk due to a combination of lower health literacy and other socio-economic or biological factors that were not directly assessed in this study. To address these disparities, it is imperative to explore the underlying causes of lower health literacy among women. Various factors, including educational background, access to healthcare services, cultural norms, and societal roles, may significantly influence the health literacy of older women [56]. Understanding these factors can help in designing interventions that are culturally and contextually appropriate. Due to the existence of sex differences, this study suggests that sex-specific health education enhancement programs can be established, such as providing paper-based health information for older men (because of their high level of education) and audio-visual videos for older women for health education. Particular attention should be paid to improving older women’s health literacy, as they have low health literacy and a high risk of falling, and improving older women’s health literacy may help to close the sex inequality in falls. We encourage policymakers to incorporate a gender perspective into health policies, ensuring that health literacy programs are both funded and specifically tailored to meet the unique needs of women. Additionally, governments should implement community-driven health literacy programs, which could focus on practical skills for disease prevention and health maintenance [57]. Organizing workshops specifically targeting women may also be a viable strategy [58], educating older women on understanding medical terminology, navigating health systems, and recognizing symptoms of common diseases.

Our study has some strengths. First, this study is the first to explore the relationship between health literacy and falls based on a community-based sample of older people in LMICs, providing important scientific evidence for falls prevention in LMICs. Second, we used a specialized scale to measure health literacy in older people and controlled for a range of potential covariates to be able to more clearly identify the relationship between health literacy and falls. Finally, we also conducted sensitivity analyses to ensure the robustness of our findings. Moreover, there are several limitations of this study. First, the cross-sectional study design did not allow for the inference of a causal relationship between health literacy and falls, and future experimental studies are needed to explore and validate causality. Second, fall-related risk factors are very diverse, and we did not include physical measurements, diet-related indicators, the history of falls and related medical conditions (stroke, fractures, Parkinson’s disease, etc.) in covariates due to data limitations, which may have confounding bias. Third, both health literacy and falls were measured using self-reporting, which may produce recall and measurement bias. Fourth, our findings are based on community-based older adults in only one region of China, and the applicability of the findings to older adults in other regions or living in institutionalized settings needs to be verified.

## Conclusions

Older people with adequate health literacy have lower prevalence of falls, which appears to differ by sex. This relationship was significant among men but not among women. These findings emphasize the need for policymakers and healthcare providers to consider sex differences when designing and implementing programs aimed at improving health literacy and preventing falls in the older population. Improving health literacy among older women could be a strategic component in bridging sex inequality in falls.

### Electronic supplementary material

Below is the link to the electronic supplementary material.

Supplementary Material 1

## Data Availability

The datasets generated during and/or analyzed during the current study are available from the corresponding author on reasonable request.
