# Supplementary material for: Health literacy and falls among community-dwelling older people in China: is there a sex difference?
Source: Aging Clin Exp Res. 2024 Jul 18;36(1):148. doi: 10.1007/s40520-024-02788-6 (PMC11258050; doi:10.1007/s40520-024-02788-6)
Supplement: Supplementary file 1 — Supplementary Material 1 [file 40520_2024_2788_MOESM1_ESM.docx]

**Health literacy and falls among community-dwelling older people in China: Is there a sex difference?**

**supplementary materials**

**1. Sample recruitment and survey administration**

A multistage, stratified cluster random sampling method was used to select participants. In the first stage, each district and county in Jinan city were divided into three layers, high, middle, and low, according to the annual per capita gross domestic product level. Two districts or counties were randomly selected from each layer. In the second stage, two towns or streets were randomly selected from the six randomly selected districts or counties, totaling 12 streets or towns. In the third stage, all older adults in two communities or administrative villages were randomly selected from the 12 randomly selected streets or towns, totaling 24 communities or administrative villages.

After identifying the selected communities or administrative villages at the sampling stage, we contacted the community or administrative village staff by telephone to introduce them to the content, purpose, and value of the study. After receiving their support, our investigator went to the community or administrative village for an enrolled survey. The investigators consisted of uniformly trained medical undergraduate students. Community or administrative village staff took the investigators into the homes of older adults to conduct paper-based questionnaires with those older adults who met the criteria. All surveys were conducted in an interview style, in which the investigator read the questions, the participants verbally responded, and then the investigator filled in the questionnaire.

**2. 10-items of health literacy screening scale**

Supplementary Table 1 10-items of health literacy screening scale

| Items | strongly disagree | disagree | neutral | agree | strongly agree |
| --- | --- | --- | --- | --- | --- |
| 1. I am capable of finding the health information that I need | 1 | 2 | 3 | 4 | 5 |
| 2. I am capable of reading health information | 1 | 2 | 3 | 4 | 5 |
| 3. I am capable of explaining the health information that I have learned | 1 | 2 | 3 | 4 | 5 |
| 4. I am capable of becoming aware of inconsistent health information | 1 | 2 | 3 | 4 | 5 |
| 5. I am capable of selecting the health information that I need | 1 | 2 | 3 | 4 | 5 |
| 6. I am capable of judging the accuracy of health information | 1 | 2 | 3 | 4 | 5 |
| 7. I am capable of describing my health problems to medical staff members such as physicians | 1 | 2 | 3 | 4 | 5 |
| 8. I am capable of sharing or communicating the health information I have learned to others | 1 | 2 | 3 | 4 | 5 |
| 9. I am capable of completing medical forms in a hospital independently | 1 | 2 | 3 | 4 | 5 |
| 10. I am capable of directing myself to the medical department to which I should go in a hospital | 1 | 2 | 3 | 4 | 5 |

**3. The measurement of depressive symptom, mild cognitive impairment, and self-rated balance impairment**

Depressive symptoms were assessed using the 15-item Geriatric Depression Scale (GDS)(3). Each item included two options: no (0 points) and yes (1 point), and the total score ranged from 0 to 15. Based on the validation results of the previous Chinese version(4), a score ≥8 indicated depressive symptoms (0=yes, 1=no).

Mild cognitive impairment was evaluated using the Mini-Mental State Examination (MMSE)(1). The MMSE includes 30 items assessing orientation, memory, attention and calculation, recall, and verbal ability. The total MMSE scores ranged from 0 to 30 points. According to a previous Chinese study (2), we determined MCI based on the MMSE score cutoffs for different education levels. Specifically, we judged illiteracy ≤20, primary school ≤24, and middle school and above ≤27 as MCI (0=yes, 1=no).

Balance impairment was evaluated using self-assessed balance ability. Participants were asked " How do you think of your balance ability??" Answers included very good, good, fair, poor, and very poor. In this study, we determined poor and very poor as having self-rated balance impairment.

**4. The health literacy score and subitem scores by sex**

Supplementary Table 2 shows the health literacy score and subitem scoring by sex. We used t-test to examine the sex difference of the health literacy score and subitem scores, and the results showed men’s scores are all higher than women’s (*P*<0.05).

Supplementary Table 2 Health literacy score and subitem scores by sex

| Health literacy | Score | Men | Women | *P* |
| --- | --- | --- | --- | --- |
| Total | 32.32±8.29 | 33.90±7.78 | 30.74±8.50 | <0.001 |
| Item 1 | 3.23±0.96 | 3.32±0.92 | 3.14±0.99 | <0.001 |
| Item 2 | 3.22±1.10 | 3.43±0.99 | 3.01±1.16 | <0.001 |
| Item 3 | 3.09±1.04 | 3.25±1.00 | 2.93±1.05 | <0.001 |
| Item 4 | 3.21±0.98 | 3.35±0.93 | 3.06±1.02 | <0.001 |
| Item 5 | 3.27±0.99 | 3.43±0.92 | 3.11±1.03 | <0.001 |
| Item 6 | 3.17±0.98 | 3.35±0.94 | 2.99±1.00 | <0.001 |
| Item 7 | 3.62±0.86 | 3.68±0.83 | 3.56±0.88 | 0.001 |
| Item 8 | 3.39±0.97 | 3.49±0.92 | 3.29±1.00 | <0.001 |
| Item 9 | 3.04±1.15 | 3.30±1.07 | 2.78±1.17 | <0.001 |
| Item 10 | 3.08±1.10 | 3.29±1.02 | 2.87±1.13 | <0.001 |

**Reference**

1. Folstein MF, Folstein SE, McHugh PR. "Mini-mental state". A practical method for grading the cognitive state of patients for the clinician. Journal of psychiatric research. 1975 Nov;12(3):189-98.

2. Cui G, Li S, Kong Q, Yin Y, Chen L, Chen L, Liu X. Association of sleep quality, depressive symptoms and their interaction with cognitive frailty in elderly people. Chinese General Practice. 2021;24(9):1076-1081.

3. Brown LM, Schinka JA. Development and initial validation of a 15‐item informant version of the Geriatric Depression Scale. International Journal of Geriatric Psychiatry: A journal of the psychiatry of late life and allied sciences. 2005;20(10):911-918.

4. Liu C-Y, Lu C-H, Yu S, Yang Y-Y. Correlations between scores on Chinese versions of long and short forms of the Geriatric Depression Scale among elderly Chinese. Psychological reports. 1998;82(1):211-214.
